# Supplementary figures and images for: Adrenomedullin restores the human cortical interneurons migration defects induced by hypoxia
Source: eLife. 2026 May 15;14:RP108134. doi: 10.7554/eLife.108134 (PMC13179061; doi:10.7554/eLife.108134)

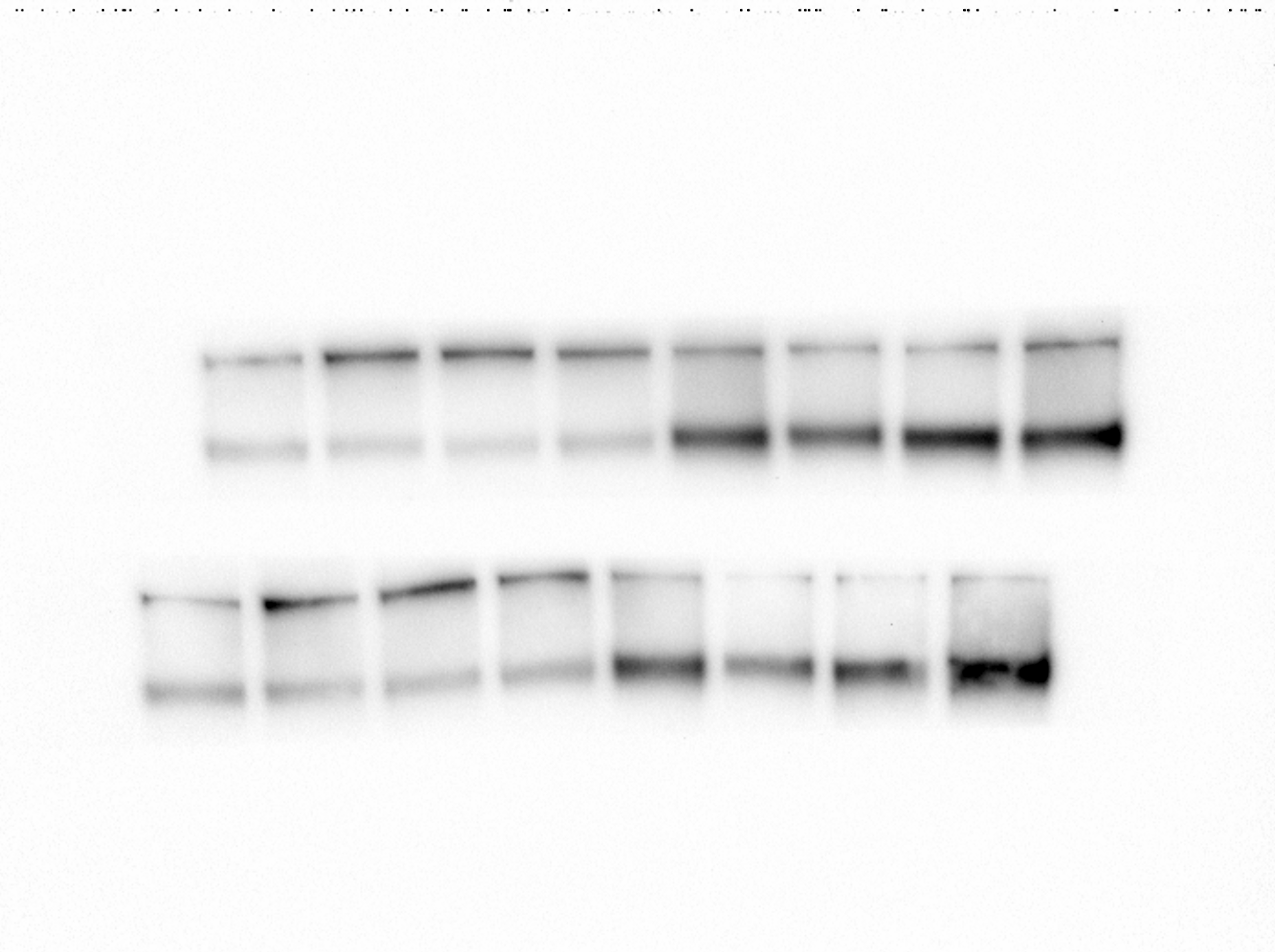

Supplement: Figure 1—figure supplement 1—source data 1. [file elife-108134-fig1-figsupp1-data1.zip › Figure 1-figure supplement 1-source data 1/2_22_22_INt9_HIF1ALPHABlotab2.tif]

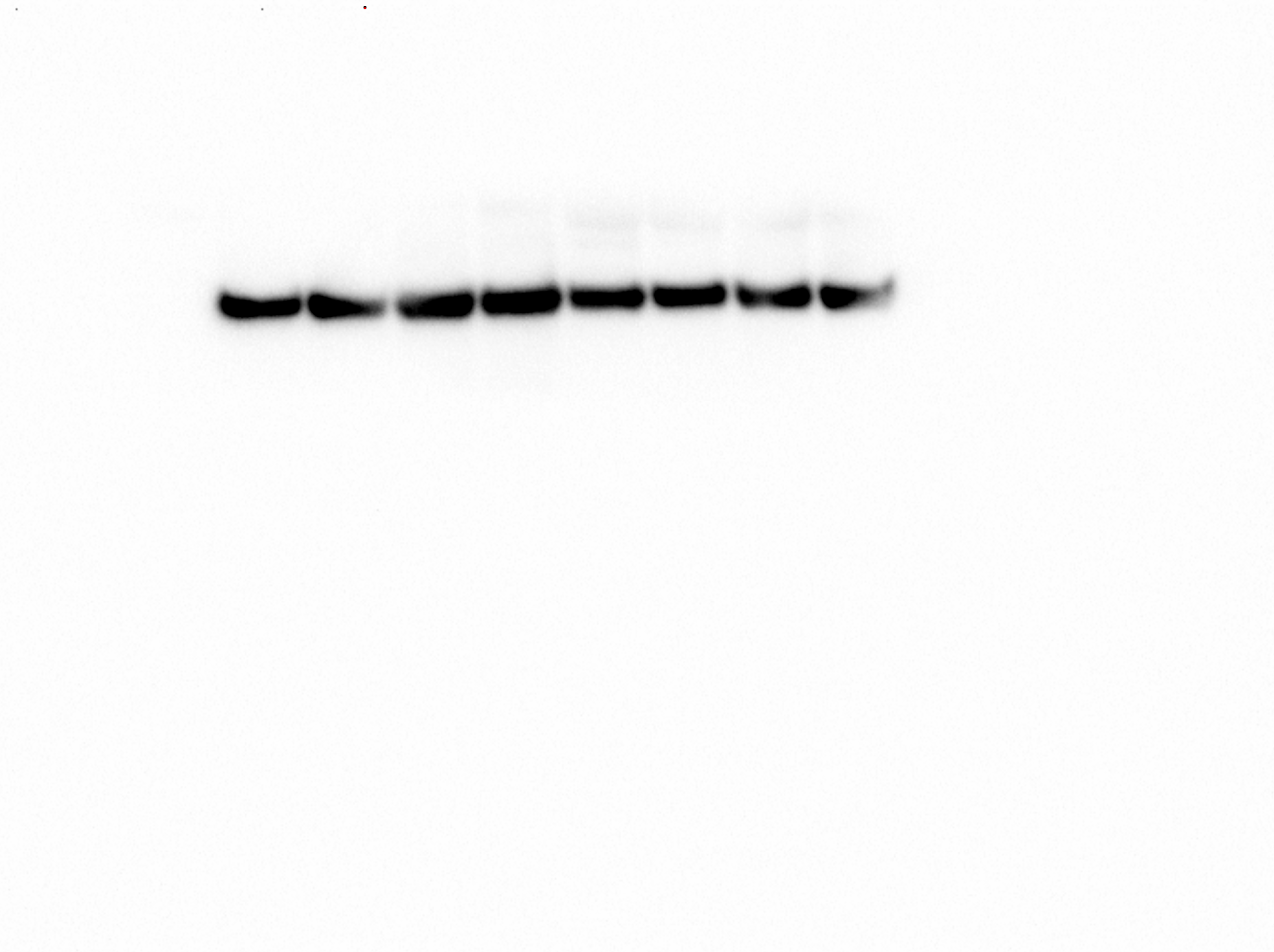

Supplement: Figure 1—figure supplement 1—source data 1. [file elife-108134-fig1-figsupp1-data1.zip › Figure 1-figure supplement 1-source data 1/3_1_22_BetaActin_BlotA_INT9.tif]

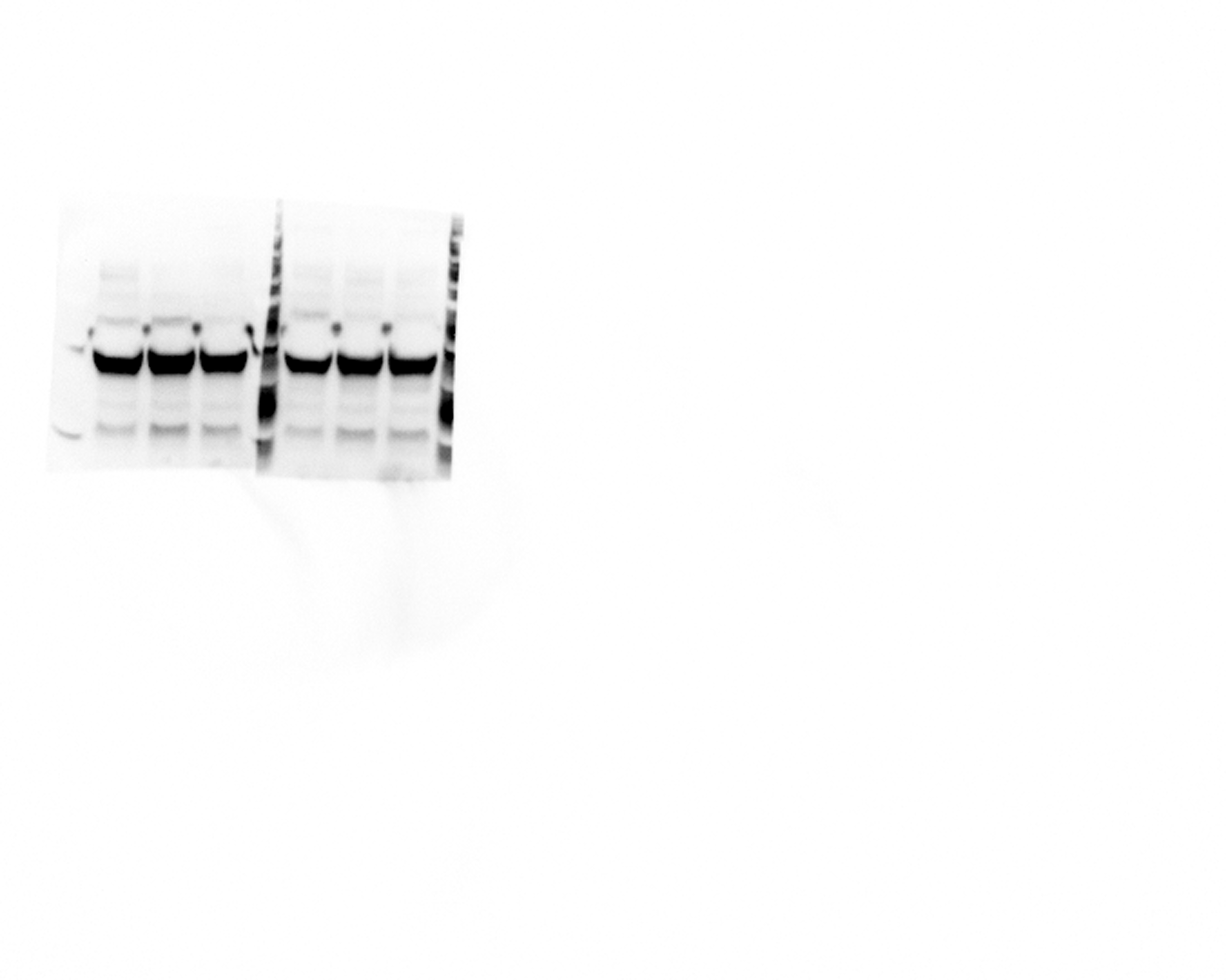

Supplement: Figure 3—source data 1. [file elife-108134-fig3-data1.zip › CHEMI_11082023_151411_(Chemi) tubulin.tif]

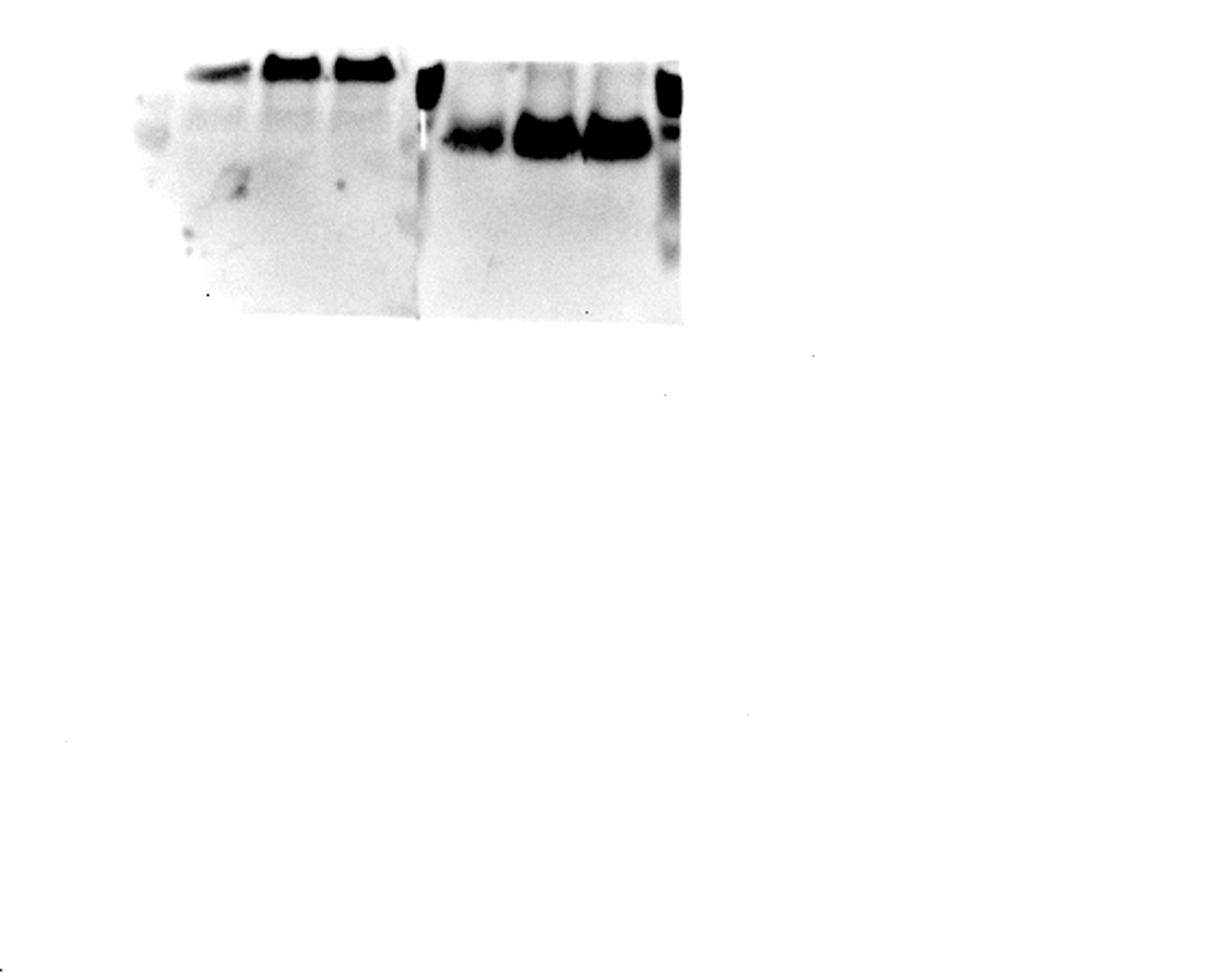

Supplement: Figure 3—source data 1. [file elife-108134-fig3-data1.zip › CHEMI_11082023_154035_(Chemi) RAMP1 RAMP2.tif]

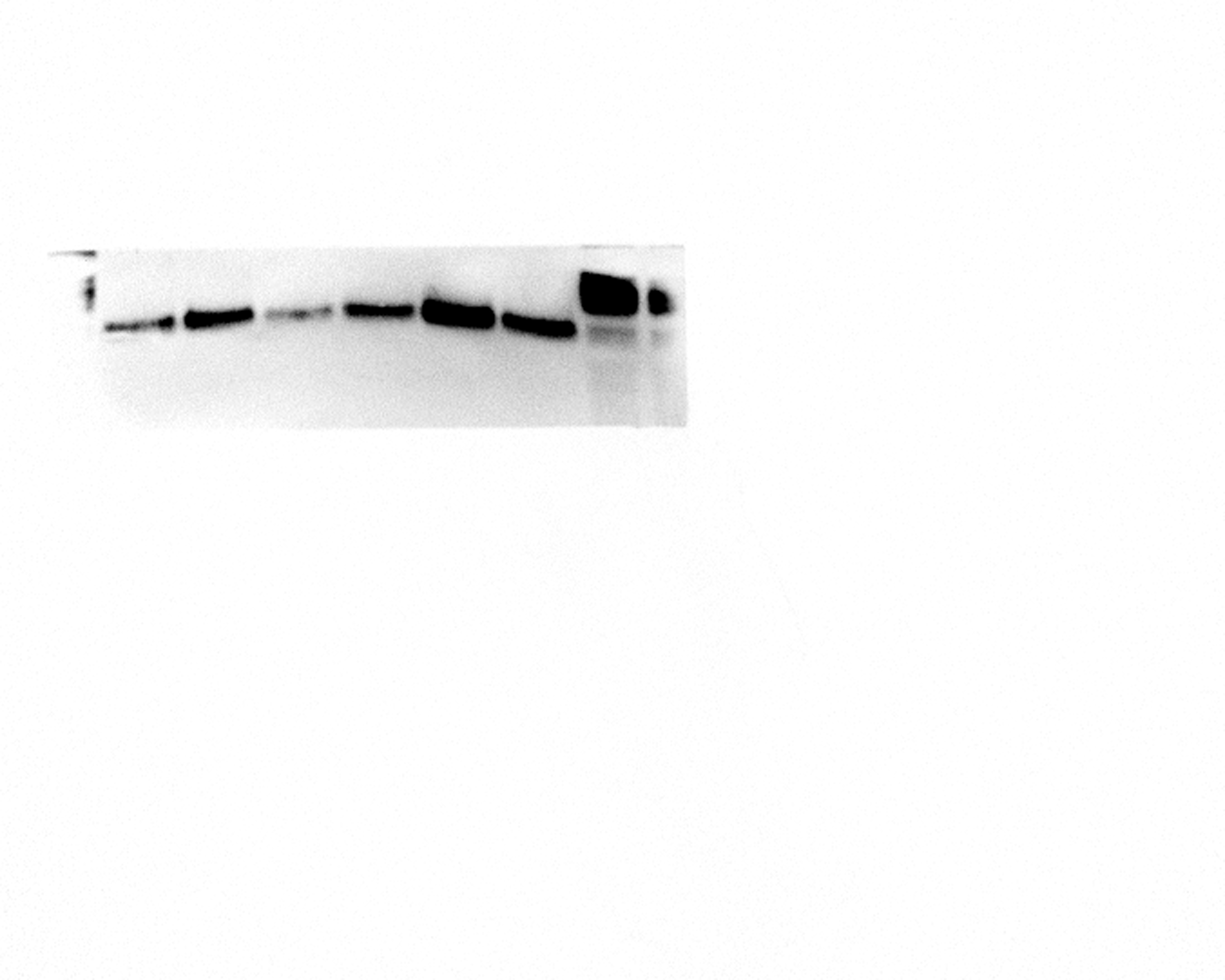

Supplement: Figure 3—source data 1. [file elife-108134-fig3-data1.zip › CHEMI_11152023_143217_(Chemi RAMP2).tif]

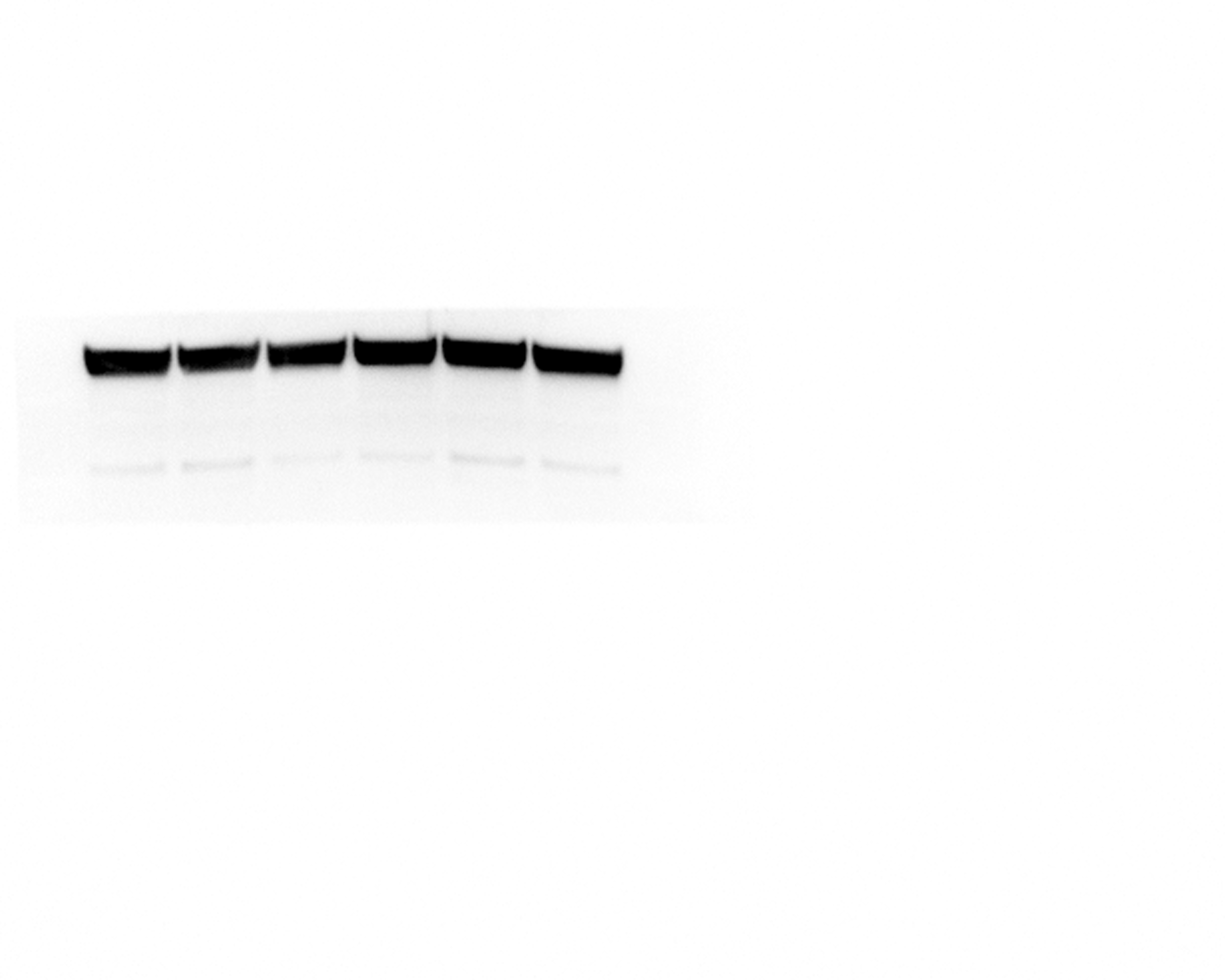

Supplement: Figure 3—source data 1. [file elife-108134-fig3-data1.zip › CHEMI_11152023_143603_(Chemi tubulin).tif]
