# Supplementary material for: Adrenomedullin restores the human cortical interneurons migration defects induced by hypoxia
Source: eLife. 2026 May 15;14:RP108134. doi: 10.7554/eLife.108134 (PMC13179061; doi:10.7554/eLife.108134)

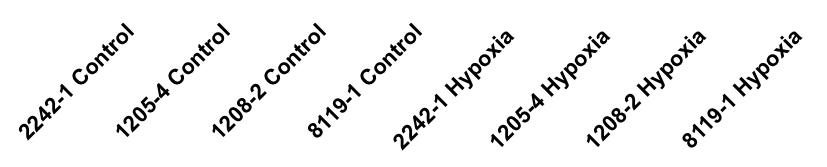


**A**


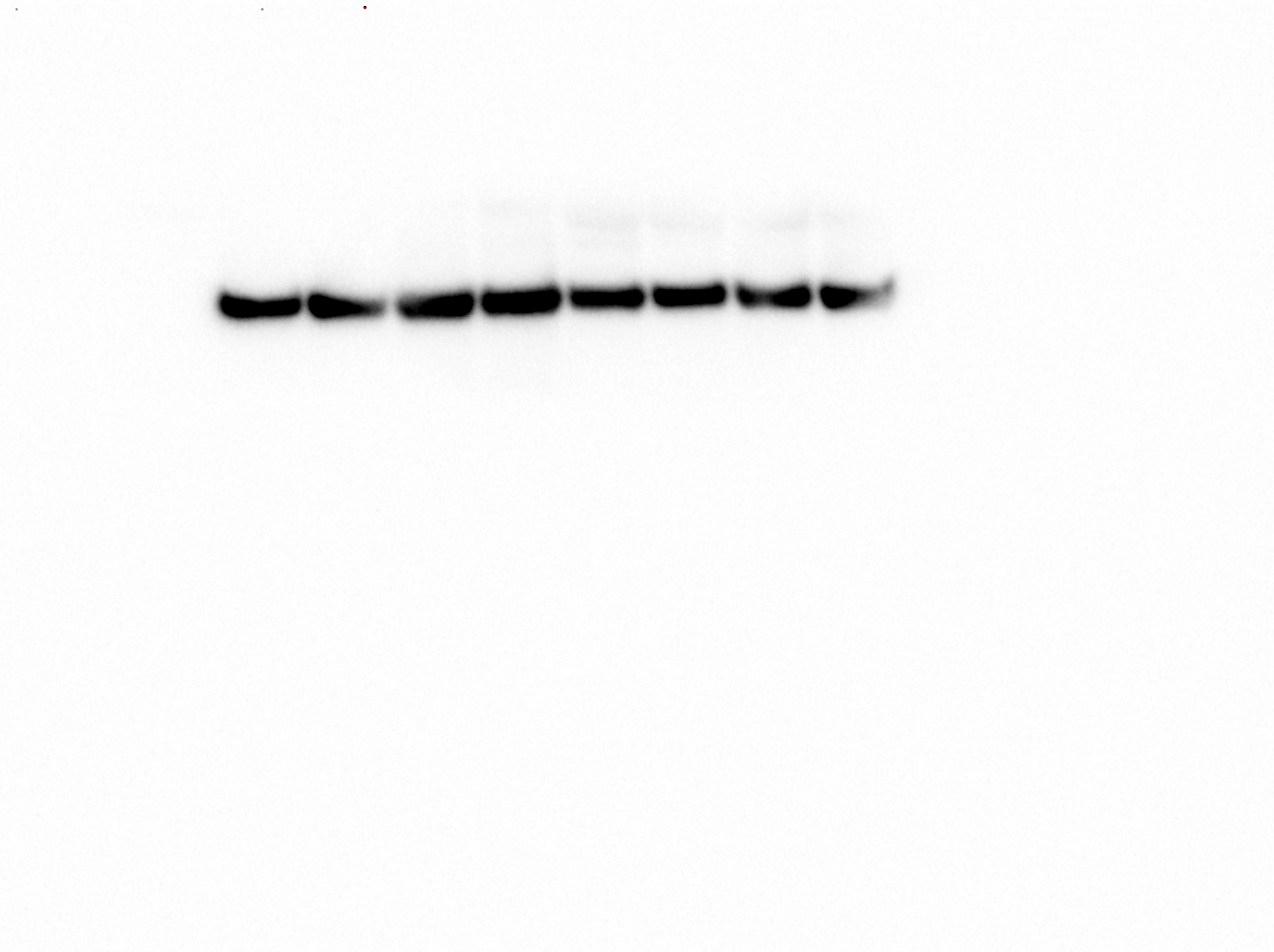


**42kDa**

**Beta-actin**


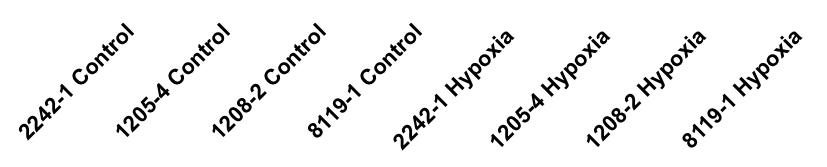


**Figure 1-supplemental figure 1C. A.** Original uncropped beta actin membrane corresponding to Figure 1, panel C., **B.** Original uncropped HIF1-Alpha membrane corresponding Figure 1, panel, C.

**100kDa**

**HIF1-Alpha**

**B**


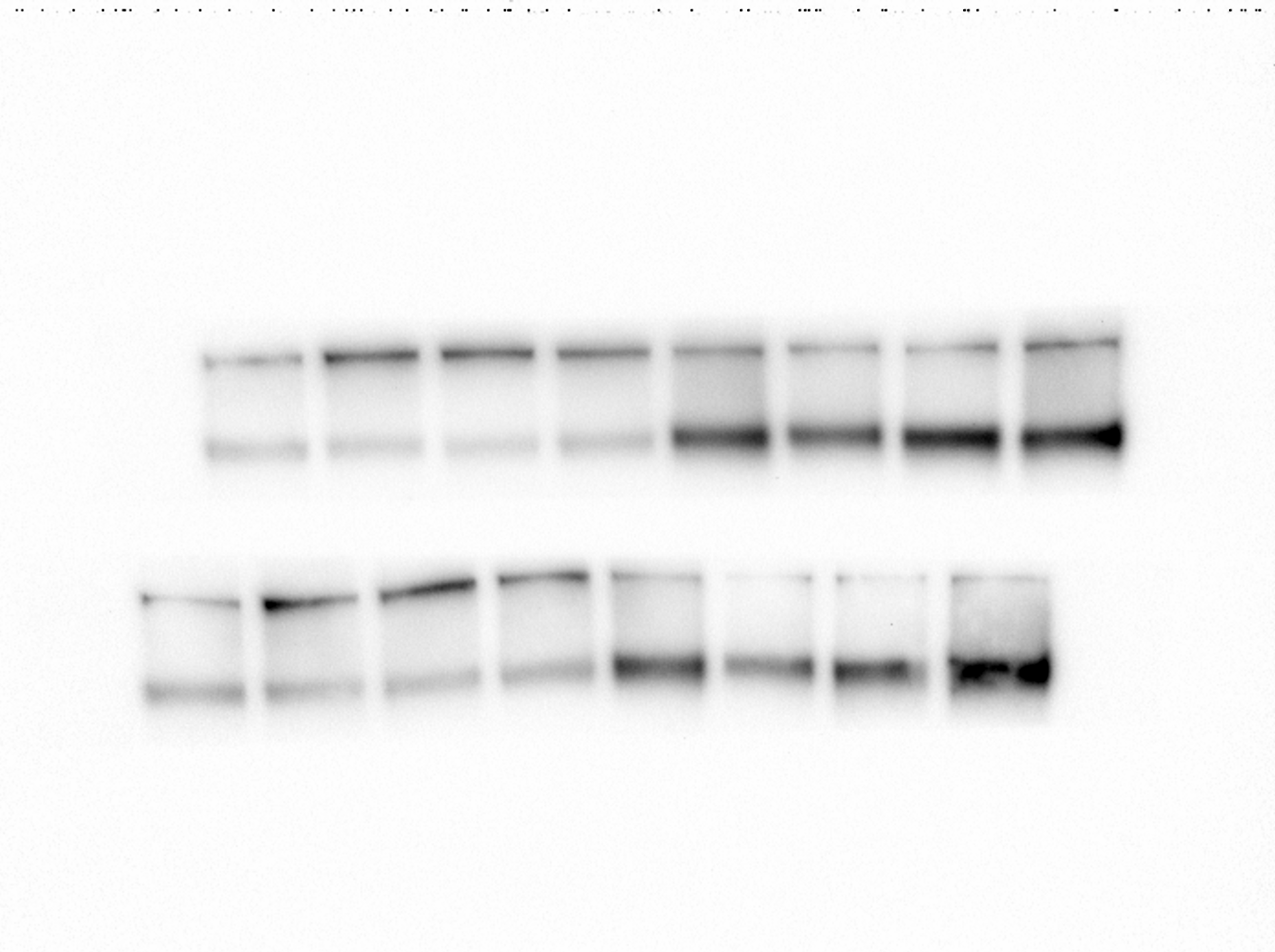

Supplement: Figure 1—figure supplement 1—source data 2. [file elife-108134-fig1-figsupp1-data2.zip › Figure 1-figure supplement 1-source data 2 .docx]
