# Supplementary material for: Adrenomedullin restores the human cortical interneurons migration defects induced by hypoxia
Source: eLife. 2026 May 15;14:RP108134. doi: 10.7554/eLife.108134 (PMC13179061; doi:10.7554/eLife.108134)
Supplement: Figure 3—source data 2. [file elife-108134-fig3-data2.zip › Figure 3H - Source data 2.docx]

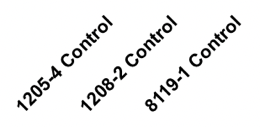

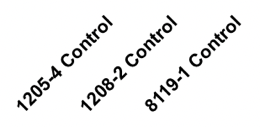

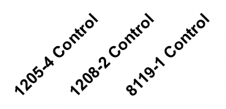

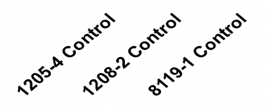

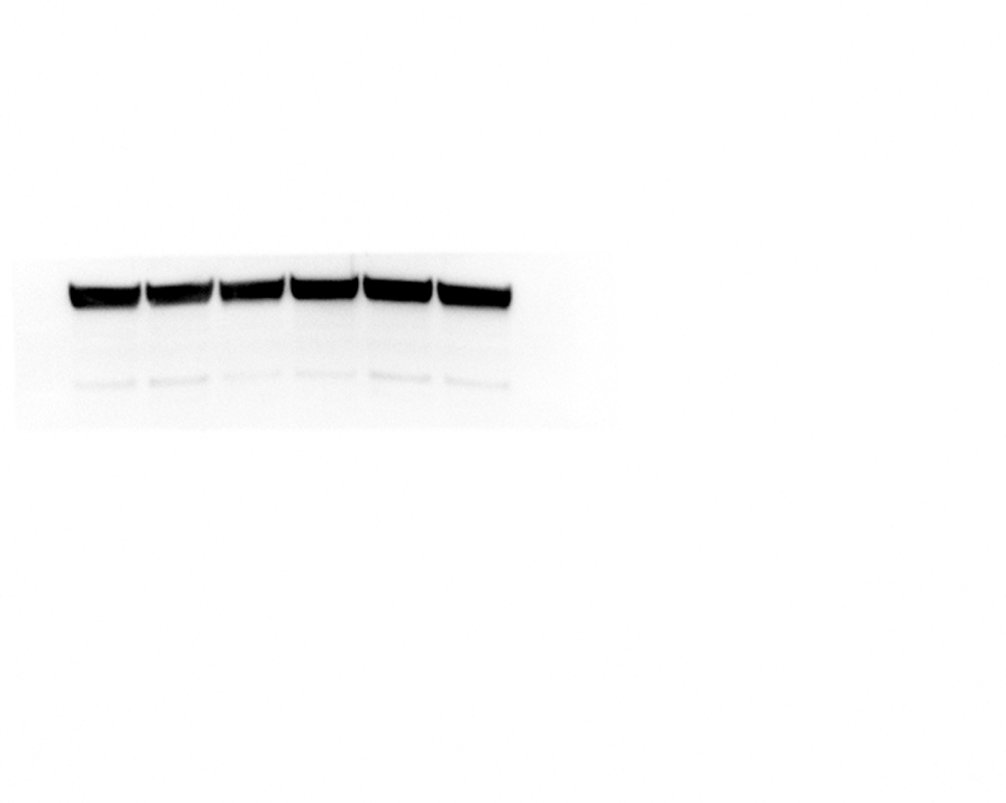

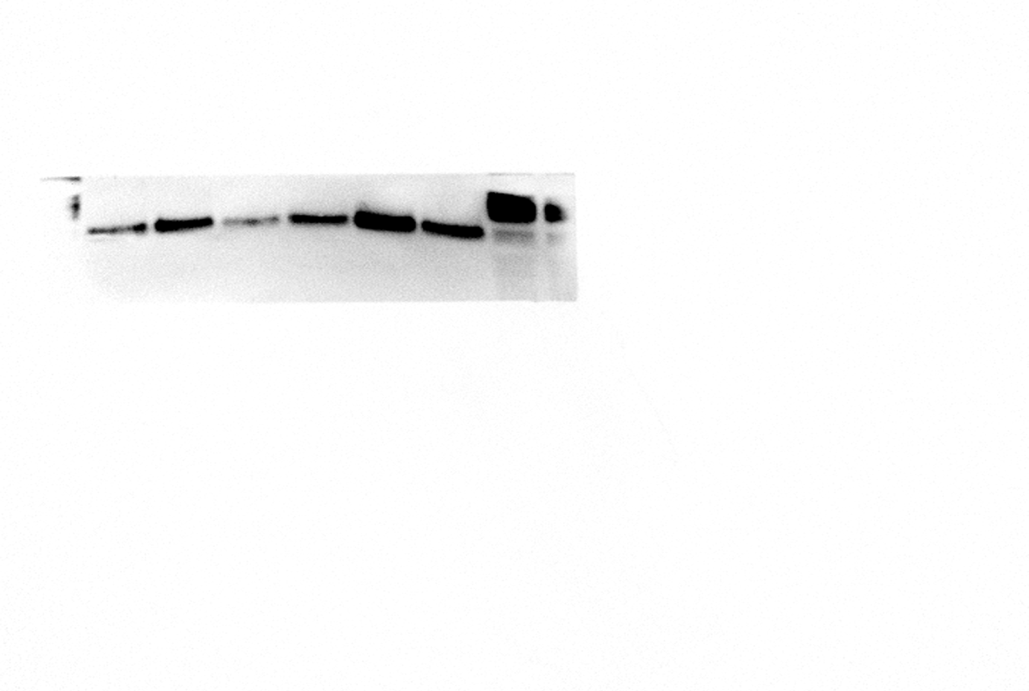

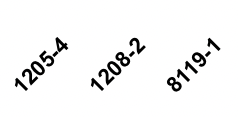

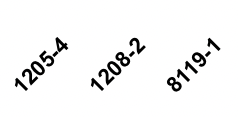


**17kDA**

**RAMP2**

**Figure 3H- source data 1. A.** Original uncropped beta tubulin membrane corresponding to Figure 3, panel H, **B.** Original uncropped RAMP2 membrane corresponding Figure 3, panel, H.

**B**

**A**

**α-tubulin**

**50kda**

**Figure 3F, source data 2**

α-tubulin

50kda
