# Supplementary material for: Adrenomedullin restores the human cortical interneurons migration defects induced by hypoxia
Source: eLife. 2026 May 15;14:RP108134. doi: 10.7554/eLife.108134 (PMC13179061; doi:10.7554/eLife.108134)
Supplement: Figure 3—source data 2. [file elife-108134-fig3-data2.zip › Figure 3F- Source data 2.docx]

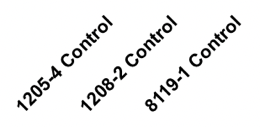

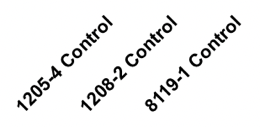

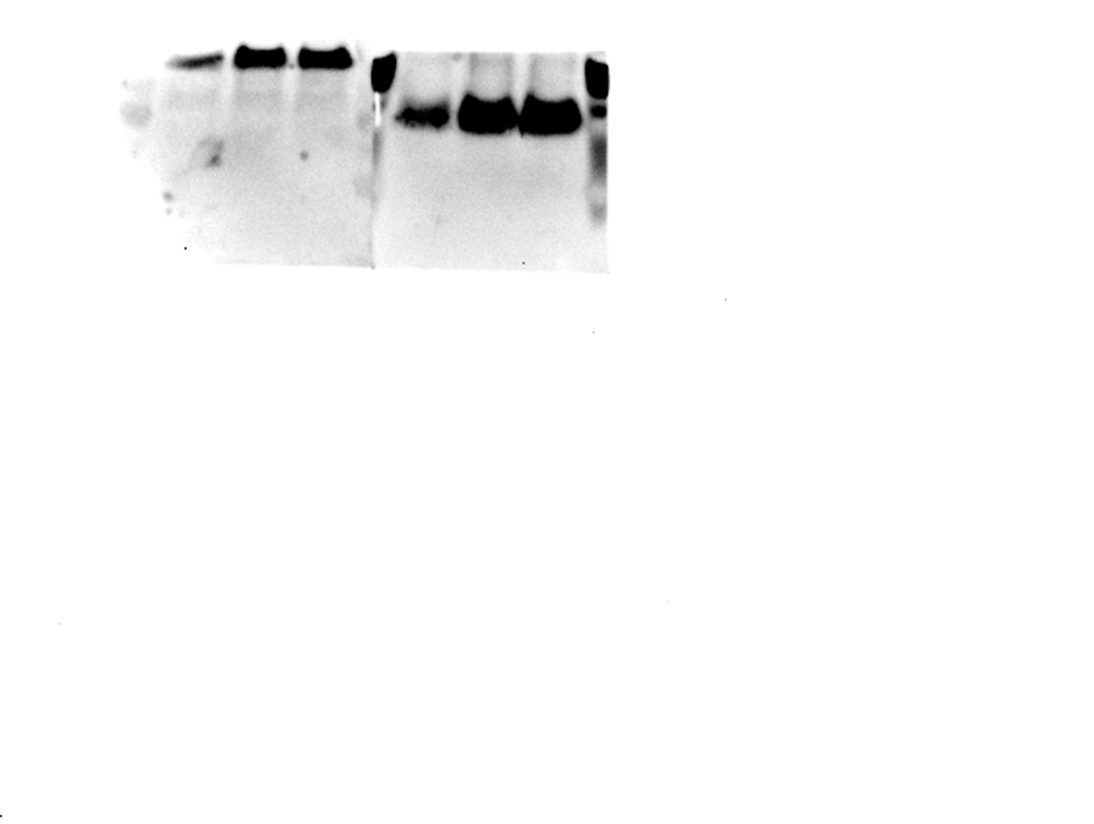

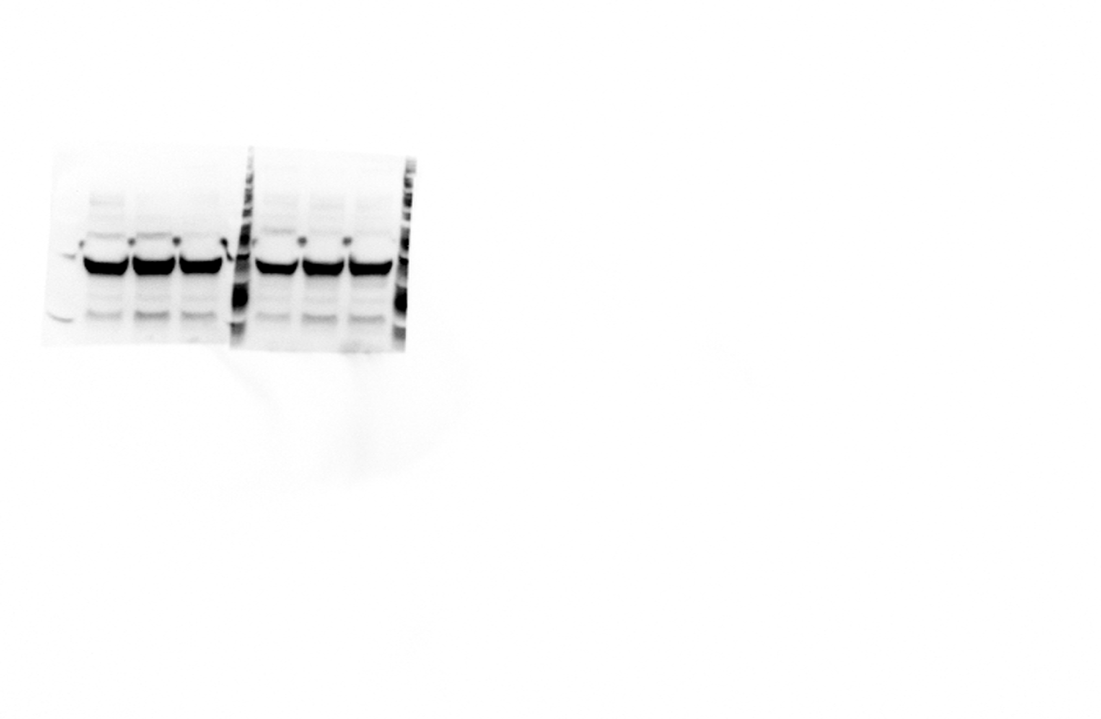

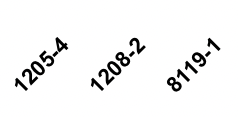

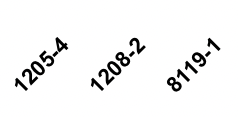

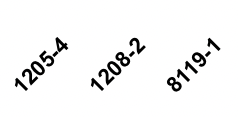

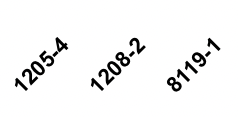


**50kda**

**α-tubulin**

**17kda**

**RAMP2**

**20kda**

**RAMP1**

**A**

**Figure 3F, source data 2. A.** Original uncropped beta tubulin membrane corresponding to Figure 3, panel F, **B.** Original uncropped RAMP1 and RAMP2 membranes corresponding Figure 3, panel, F.

**B**

**Figure 3F, source data 2**

α-tubulin

50kda
