# Supplementary material for: Adrenomedullin restores the human cortical interneurons migration defects induced by hypoxia
Source: eLife. 2026 May 15;14:RP108134. doi: 10.7554/eLife.108134 (PMC13179061; doi:10.7554/eLife.108134)
Supplement: Supplementary file 4. [file elife-108134-supp4.docx]

| ENSG00000274956 | NKAIN3-IT1 | lncRNA | -1.284747906 | 0.006164441 | 0.000296038 | 47.67559988 |
| --- | --- | --- | --- | --- | --- | --- |
| ENSG00000221630 | MIR1179 | miRNA | -1.310660901 | 0.030037406 | 0.002829342 | 9.099708689 |
| ENSG00000135824 | RGS8 | protein_coding | -1.322336212 | 0.002215407 | 7.38E-05 | 34.42000216 |
| ENSG00000183908 | LRRC55 | protein_coding | -1.338432053 | 0.000122127 | 2.03E-06 | 60.70122131 |
| ENSG00000188517 | COL25A1 | protein_coding | -1.340485402 | 0.008773344 | 0.000499224 | 18.16934139 |
| ENSG00000198883 | PNMA5 | protein_coding | -1.347521594 | 0.019646352 | 0.001536373 | 11.61837614 |
| ENSG00000210117 | MT-TW | Mt_tRNA | -1.446983788 | 0.017591752 | 0.001307943 | 15.72187853 |
| ENSG00000116254 | CHD5 | protein_coding | -1.468744699 | 0.007946962 | 0.000430726 | 38.75447678 |
| ENSG00000198840 | MT-ND3 | protein_coding | -1.495528323 | 5.46E-17 | 5.06E-20 | 2158.272751 |
| ENSG00000198948 | MFAP3L | protein_coding | -1.516941186 | 0.000775047 | 1.90E-05 | 9.700905667 |
| ENSG00000112038 | OPRM1 | protein_coding | -1.544068897 | 0.028075389 | 0.002574168 | 13.13781863 |
| ENSG00000174145 | NWD2 | protein_coding | -1.546376715 | 0.023170282 | 0.001960672 | 8.845361683 |
| ENSG00000198400 | NTRK1 | protein_coding | -1.54977278 | 0.02043442 | 0.001619897 | 25.9926564 |
| ENSG00000162595 | DIRAS3 | protein_coding | -1.552293505 | 0.003805426 | 0.000153663 | 30.34159846 |
| ENSG00000248527 | MTATP6P1 | unprocessed_pseudogene | -1.57616242 | 0.000964872 | 2.50E-05 | 291.6307677 |
| ENSG00000163285 | GABRG1 | protein_coding | -1.603820761 | 0.021612573 | 0.001767118 | 11.03080557 |
| ENSG00000027644 | INSRR | protein_coding | -1.665485595 | 0.011029592 | 0.000682963 | 34.30693968 |
| ENSG00000168959 | GRM5 | protein_coding | -1.689507319 | 0.012229777 | 0.000792206 | 35.346747 |
| ENSG00000255836 | AC131206.1 | processed_pseudogene | -1.690231302 | 0.01271025 | 0.000837079 | 11.05531427 |
| ENSG00000286214 | AUXG01000058.1 | lncRNA | -1.724269907 | 0.007130638 | 0.000367212 | 674.2804126 |
| ENSG00000117152 | RGS4 | protein_coding | -2.004797365 | 6.58E-05 | 9.90E-07 | 16.75346885 |
| ENSG00000210195 | MT-TT | Mt_tRNA | -2.222042025 | 1.50E-05 | 1.78E-07 | 9.639353259 |
| ENSG00000079689 | SCGN | protein_coding | -2.274450893 | 0.017067983 | 0.001257151 | 11.98765259 |
| ENSG00000210196 | MT-TP | Mt_tRNA | -2.391985922 | 1.48E-16 | 1.60E-19 | 61.98408144 |
| ENSG00000198695 | MT-ND6 | protein_coding | -2.542733456 | 2.36E-22 | 1.82E-26 | 369.7526992 |
| ENSG00000210107 | MT-TQ | Mt_tRNA | -3.191895797 | 2.24E-07 | 1.94E-09 | 14.722918 |
